# Relationship between sclerostin and cardiovascular calcification in hemodialysis patients: a cross-sectional study

**DOI:** 10.1186/1471-2369-14-219

**Published:** 2013-10-10

**Authors:** Vincent M Brandenburg, Rafael Kramann, Ralf Koos, Thilo Krüger, Leon Schurgers, Georg Mühlenbruch, Sinah Hübner, Ulrich Gladziwa, Christiane Drechsler, Markus Ketteler

**Affiliations:** 1 Department of Cardiology, University Hospital of the RWTH, Pauwelsstraße 30, D- 52057 Aachen, Germany; 2 Department of Nephrology, University Hospital of the RWTH Aachen, Aachen, Germany; 3 Department of Biochemistry, Cardiovascular Research Institute Maastricht (CARIM), Maastricht University, Maastricht, the Netherlands; 4 Department of Neuro-Radiology, University Hospital of the RWTH Aachen, Aachen, Germany; 5 Dialysis Center, Kuratorium für Heimdialyse, Würselen, Germany; 6 Department of Internal Medicine 1, Division of Nephrology, University of Würzburg, Würzburg, Germany; 7 Department of Nephrology, Klinikum Coburg, Coburg, Germany

**Keywords:** Aortic valve disease, Cardiovascular disease, Coronary calcification, Hemodialysis, Mineral metabolism, Vascular calcification, Renal osteodystrophy, Sclerostin

## Abstract

**Background:**

Sclerostin is a Wnt pathway antagonist regulating osteoblast activity and bone turnover. Here, we assessed the potential association of sclerostin with the development of coronary artery (CAC) and aortic valve calcifications (AVC) in haemodialysis (HD) patients.

**Methods:**

We conducted a cross-sectional multi-slice computed tomography (MS-CT) scanning study in 67 chronic HD patients (59.4 ± 14.8 yrs) for measurement of CAC and AVC. We tested established biomarkers as well as serum sclerostin (ELISA) regarding their association to the presence of calcification. Fifty-four adults without relevant renal disease served as controls for serum sclerostin levels. Additionally, sclerostin expression in explanted aortic valves from 15 dialysis patients was analysed *ex vivo* by immunohistochemistry and mRNA quantification (Qt-RT-PCR).

**Results:**

CAC (Agatston score > 100) and any AVC were present in 65% and in 40% of the MS-CT patient group, respectively. Serum sclerostin levels (1.53 ± 0.81 vs 0.76 ± 0.31 ng/mL, p < 0.001) were significantly elevated in HD compared to controls and more so in HD patients with AVC versus those without AVC (1.78 ± 0.84 vs 1.35 ± 0.73 ng/mL, p = 0.02). Multivariable regression analysis for AVC revealed significant associations with higher serum sclerostin. *Ex vivo* analysis of uraemic calcified aortic valves (*n* = 10) revealed a strong sclerostin expression very close to calcified regions (no sclerostin staining in non-calcified valves). Correspondingly, we observed a highly significant upregulation of sclerostin mRNA in calcified valves compared to non-calcified control valves.

**Conclusion:**

We found a strong association of sclerostin with calcifying aortic heart valve disease in haemodialysis patients. Sclerostin is locally produced in aortic valve tissue adjacent to areas of calcification.

## Background

The majority of long-term haemodialysis (HD) patients are affected by cardiovascular calcification (CVC) [1,2]. CVC constitutes one of the driving forces for the enormously elevated cardiovascular mortality in patients with CKD or end-stage renal disease (ESRD) [3,4].

Numerous proteins have been identified to be significantly associated with the presence and amount of uraemic CVC in the past (e.g. osteoprotegerin (OPG), alkaline phosphatase (AP), fetuin-A, matrix-gla protein (MGP), parathyroid hormone (PTH), C-reactive protein (CRP)) [4-8], some of which are also associated with morbidity and mortality in dialysis patients [9-11]. Several of these parameters are also involved in the regulation of bone metabolism and osseous calcification processes. Immunohistochemistry examinations of calcified cardiovascular structures showed that some of these “bone proteins” are deposited adjacent to calcification nidus indicating an osseous transdifferention of the vascular wall cells [12].

A novel candidate protein for this bone-vascular axis is sclerostin (22.5 kDa), which is synthesized in osteocytes and is a potent down-regulator of bone metabolism by reducing osteoblast differentiation and function via canonical Wnt-signalling inhibition [13,14]. High serum sclerostin was described in patients with CKD [15]. Recent reports indicated that serum sclerostin levels may reflect reduced bone metabolism and may be useful as marker for low-turnover bone disease in ESRD patient [16]. Therefore, we hypothesized that sclerostin is also linked to vascular calcification in dialysis patients.

The aim of the present study was to assess whether CVC in haemodialysis patients is associated with levels of circulating sclerostin and whether valvular calcification goes along with local sclerostin production. We performed a cross-sectional study among HD patients who underwent MS-CT scanning for CAC and AVC assessment. Additionally, an *ex vivo* study with aortic valve tissue samples was performed in order to analyse local valvular sclerostin expression in calcified versus non-calcified aortic valve tissue.

## Methods

### Patient characteristics

**Figure 1:**
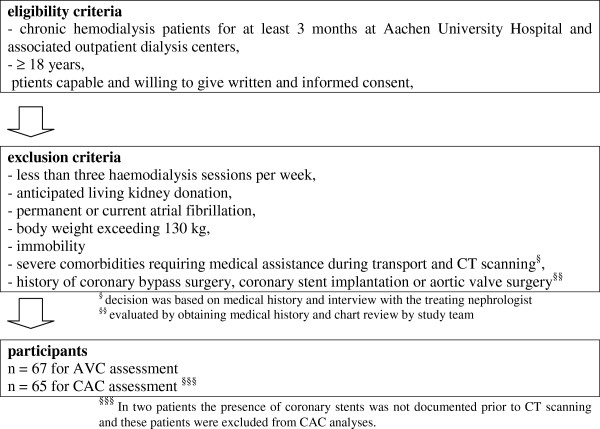
Flow diagram indicating detailed list of eligibility criteria and exclusion criteria of MSCT patients.

Patients for MSCT calcification assessment were all chronic HD patients. Standard bicarbonate dialysis procedures were thrice weekly haemodialysis or haemodiafiltration sessions (4.5 to 5.5 hrs). Dialysate calcium concentration was 1.25 or 1.5 mmol/L. All adult hemodialysis patients from the Aachen University Hospital and three collaborating dialysis centers were eligible after written and informed consent. Patients were not included in the study if they anticipated living kidney donation, had current atrial fibrillation, severe comorbidities, a history of coronary bypass surgery, coronary stent implantation or aortic valve surgery (patient characteristics Table 1) (Figure 1: flow diagram of a detailed list of inclusion and exclusion criteria). Two patients underwent MSCT despite prior stent implantation. These two patients were included only for AVC analysis. Twenty-one patients had a previous renal transplantation. Median interval between re-initiation of dialysis after transplant failure prior to cardiac MS-CT was 28 months (range 4 – 63 months). A subgroup of 40 patients from the entire MS-CT cohort were included in a previous publication [6]. In the entire cohort of 67 patients, the dominant causes for ESRD were glomerulonephritis or systemic vasculitis in *n* = 21 (31%), ADPKD in *n* = 9 (13%), renal vascular disease or hypertensive nephropathy in *n* = 11 (16%) and diabetic nephropathy in *n* = 6 (9%). Arterial hypertension was defined as use of antihypertensive drugs or arterial blood pressure exceeding 130/85 mmHg.

**Table 1:** Clinical, demographic, and MSCT data of the entire MSCT cohort (n = 67)

		**Entire cohort (*n*** **= 67)**
Women	[n,%]	35, 52%
Median age (range)	[years]	61 (21 – 87)
Median BMI (range)	[kg/m^2^]	24 (17 – 53)
Median time ESRD (range)	[months]	49 (2 – 364)
Previous renal transplant	[n,%]	21, 31%
Current smoking	[n,%]	10, 15%
History of CVD	[n,%]	25, 37%
History of CAD	[n,%]	22, 33%
Diabetes	[n,%]	15, 22%
History of PAD	[n,%]	9, 13%
History of MI	[n,%]	9, 13%
History of PTex	[n,%]	9, 13%
Median CAC Agatston (range) (*n* = 65)		279 (0 – 3736)
Median Mass CAC (range) (*n* = 65)		51 (0 – 643)
Median Volume CAC (range) (*n* = 65)		261 (0 – 3267)
Median AVC Agatston (range)		0 (0 – 1596)
Median Mass AVC (range)		0 (0 – 284)
Median Volume AVC (range)		0 (0 – 1316)
CAC Agatston score >100	[n,%]	44, 66%
Any AVC	[n,%]	27, 40%
Usage of Phosphate binders	[n,%]	61, 92%
Calcium-based	[n,%]	51, 77%
Aluminum-based	[n,%]	14, 22%
Calcimimetics	[n,%]	16, 25%

*Abbreviations*: *AVC* aortic valve calcification; *BMI* body mass index; *CAC* coronary artery calcification; *CAD* coronary artery disease; *CVD* cerebrovascular disease; *ESRD* end-stage renal disease; *MI* myocardial infarction; *MSCT* multi-sclice computed tomography; *PAD* peripheral arterial disease; *Ptex* parathyroidectomy.

The sclerostin control group consisted of 54 patients from an internal medicine outpatient department (*n* = 15 (28%) males, mean age 57.9 ± 9.7 years, eGFR >60 mL/min (MDRD formula [17])), whose primary reason for referral was not cardiovascular disease. Those patients were recruited solely based on the absence of overt CKD and not matched to the MSCT cohort in terms of age, sex or underlying extra-renal disease. Within this cohort a broad spectrum of internal medicine diseases was present. Presence or absence of cardiovascular calcification was not systematically evaluated in this cohort, so we on purpose avoid the term “healthy” controls.

We performed *ex vivo* aortic tissue analyses from overall 15 consecutive long-term HD patients (10 with AVC, 5 without AVC) who did not participate in the MSCT study. Mean age of HD patients with AVC was 56 ± 14 years (7 men). Mean age of HD patients with non-calcified aortic valves was 59 ± 9 years (three men). Non-calcified aortic valves were identified based on negative routine von Kossa staining. All valves were paraffin embedded prior to IHC staining for sclerostin. The material was retrieved from the Aachen University Pathology Institute biobank after query for “dialysis and aortic valve”. Patients with AVC as indicated by positive van Kossa had undergone aortic valve replacement due to severe aortic valve stenosis. The patients without AVC on van Kossa staining had the clinical picture of endocarditis with aortic regurgitation prior to surgery. Based on these selection criteria via the pathology biobank, detailed clinical and laboratory data regarding pre-operative conditions and history were not available in all these patients.

This study was approved by the ethical committee of the RWTH Aachen University Hospital (ethical vote EK 239/11).

### CT imaging procedure

All MSCT examinations were performed on a 16-slice MSCT scanner (SOMATOM, Sensation 16, Siemens, Forchheim, Germany). Scan parameters included a collimation of 12 × 0.75 mm, a rotation time of 420 ms, a table feed of 3.4 mm per rotation, a tube voltage of 120 kV and an effective tube current time product of 150 mAseff. For ECG-synchronization, retrospective ECG gating was applied. Axial images were reconstructed in mid-diastole at 60% of the RR interval with an effective slice thickness of 3 mm and a reconstruction increment of 2 mm. A dedicated convolution kernel (B35f), a field of view of 180 × 180 mm^2^ and a matrix of 512 × 512 were applied. Image analysis was performed on a separate computer workstation (Leonardo, Siemens, Forchheim, Germany) equipped with a dedicated software tool for calcium scoring (Calcium Scoring CT, Siemens, Forchheim, Germany). The CT scans started cranially above the origin of the left main coronary artery and moved caudally to the level of the diaphragm to include all three coronary arteries completely. Complete scanning of the entire aortic arch was not part of the routine protocol. CAC and AVC scores were calculated as the primary read-out according to the method originally described by Agatston et al. [18]. Additionally, the results were expressed as the calcification volume score and mass score automatically provided by the CT-software.

### Biochemical analysis

The parameters serum creatinine (S-creatinine), serum urea, serum phosphorus, total serum calcium (Ca2+), haemoglobin (Hb), C-reactive protein (CRP), alkaline phosphates (AP) and albumin were all measured via standard laboratory methods. We measured total serum calcium without correction for albumin.

For centralized laboratory measurements serum was harvested after an overnight fast after the long dialysis interval prior to dialysis, according to standard procedure, and immediately frozen (-20°C). Afterwards, samples were transferred to the University Hospital Aachen for long-term storage at -80°C. The following parameters were all measured centrally after study end: undercarboxylated matrix-Gla protein (ucMGP), fetuin-A, osteoprotegerin (OPG), sclerostin, bone alkaline phosphatase (BAP), and intact parathyroid hormone (iPTH).

UcMGP was measured using a competitive ELISA as previously described by our group [19].

Commercially available ELISA assays were used to determine levels of fetuin-A, OPG, and BAP (TECOmedical AG, Sissach, Switzerland). PTH was measured as intact PTH (iPTH) by an assay provided by Biomerica, USA. Serum sclerostin was assessed by the TECO® Sclerostin EIA Kit which is a 96-well immuno-capture ELISA product. Serum samples were incubated with a biotinylated polyclonal antibody as well as with a horseradish peroxidase-labelled secondary monoclonal antibody that specifically recognizes human Sclerostin. Sclerostin molecules are captured on the plate through the binding of streptavidin to the biotinylated primary antibody. After an overnight incubation, the unbound material was washed away. After this washing step, the TMB substrate, which reacts with the HRP, was added to the well and colour was formed. After 15 minutes of incubation, the reaction was stopped with HCl and the plate was read using a plate reader at 450 nm. The amount of colour generated is directly proportional to the amount of sclerostin in the sample (intratest variability 3.5 – 5.5%).

### Analysis of sclerostin expression in aortic valves

After fixation (3.7% formaldehyde, 24 hours) the valves were paraffin-embedded, cut with a rotating microtome at 3-μm thickness (Leica), and stained according to routine histology protocols. Immunohistochemical analysis was performed using the primary antibody for sclerostin (rabbit polyclonal, 1:200, Santa Cruz Biotechnology, USA, sc-130258). Slide preparations were stained by using an autostainer for immunohistochemistry (DAKO cytomation) with the biotinylated secondary antibody (rabbit/mouse, DAKO), as described previously [20].

Total RNA was isolated from formalin-fixed paraffin-embedded aortic valves using RNeasy FFPE Kit (Qiagen, Hilden, Germany). The RNA concentration was determined by measuring absorbance at 260 nm (Nanodrop, Thermo Scientific, Wilmington, USA). One microgram of RNA was reverse transcribed using the high-capacity cDNA Reverse Transcriptase Kit (Applied Biosystems, Foster City, CA). Quantitative PCR were carried out with Power SYBR Green PCR Master Mix (Applied Biosystems) and the ABI Prism 7300 (Applied Biosystems). The expression of genes of interest was normalized against the housekeeping gene GAPDH. The cDNA of the non-calcified valves from the control patients was used as a relative standard for the sclerostin expression and relative gene expression was analysed with the 2-ΔΔCt method. The following primers were used: GAPDH Fw 5′-GAAGGTGAAGGTCGGAGTCA-3′ Rv 5′-TGGACTCCACGACGTACTCA-3′, Sclerostin Fw 5′-CCGGTTCATGGTCTTGTTGTT-3′ Rv 5′-ATGCCACGGAAATCATCCC-3′.

### Statistical analysis

All analyses were done using SAS® statistical software, V9.1.3 (SAS Institute, Cary, NC, USA). Grouping of patients by the Agatston level was used to describe the distribution of serum parameters. We expressed continuous factors as mean values and standard deviation (SD). Categorical factors were presented by frequencies and percentage. To characterize the relation between Agatston level, Pearson correlation coefficients (r) were calculated. Unpaired t-tests were used in order to determine differences between groups (e.g. patients with high versus low levels of CVC according to the Agatston level).

In order to investigate the association of various biomarkers with CAC and AVC, we studied all exploratory factors in the first instance in a univariate regression model for continuous factors or in an ANOVA model for categorical factors. Factors which showed a P-value < 0.25 in the univariate analyses were studied simultaneously in a multivariate model (ANCOVA) to explore those factors contributing significant information (P-value falling below the 5% margin) for predicting the severity of cardiovascular calcification. These analyses were done for the AVC and CAC expressed as Agatston score (primary analysis target), and mass score as well as volume score (secondary analysis targets). Regarding CAC the multivariate analyses were repeated after log transformation of CAC levels in those patients with CAC baseline levels greater than zero (n = 61). Log transformation was not performed regarding AVC levels because of the high proportion of patients without AVC calcification.

Student’s *t*-test was used for statistical analysis of sclerostin mRNA expression.

All test results are reported as P-values (P), estimates and corresponding 95% limits of confidence.

## Results

### Serum sclerostin in patients

In 54 controls serum sclerostin levels were significantly lower than in HD patients [0.76 ± 0.31 vs 1.53 ± 0.81 ng/mL (p <0.00019)]. The median (25^th^ to 75^th^ percentile) levels of sclerostin were 0.69 (0.58 to 0.89) ng/mL in controls and 1.44 (0.95 to 2.01) ng/mL in patients. In controls the distribution of sclerostin levels was as follows: 14% revealed serum sclerostin < 0.50 ng/mL, 72% between 0.51 to 1.00 ng/mL, and 14% > 1.01 ng/mL. In hemodialysis patients the distribution was broader and right-shifted with levels < 0.50 ng/mL in 7%, 0.51 to 1.00 ng/mL in 14%, 1.01 to 1.50 ng/mL in 32%, 1.51 to 2.00 ng/mL in 19%, 2.01 to 2.50 ng/mL in 14% and > 2.51 ng/mL in 14%, respectively.

### Distribution of calcification levels for CAC and AVC

There were only 6 patients (9%) without CAC and 40 patients (60%) without detectable AVC. The mean Agatston CAC score was 647 ± 854 (range 0 – 3736). 23 patients had CAC levels below 100 (35%) and 29 (45%) showed CAC scores exceeding 400. The mean CAC mass and volume score were 118 ± 155 and 572 ± 720, respectively. The mean level of AVC was 76 ± 233 (range 0 – 1596, Agatston score) at baseline. Twelve patients (18%) showed AVC Agatston score > 100. The mean AVC mass score was 13 ± 41and the AVC volume score was 64 ± 194.

### Association between CAC and AVC

There was a statistically significant correlation between CAC and AVC (r = 0.34, p = 0.0055). For patients with CAC exceeding 100, the AVC Agatston score was not significantly different from patients with CAC below 100 (104 ± 288 vs 14 ± 35, p = 0.143). Accordingly, patients positive for AVC at baseline did not have significantly different CAC scores (870 ± 915 vs 507 ± 794 for patients with no AV; p = 0.095).

### Intergroup comparison between patients with different levels of calcification

After stratifying the patients for CAC < 100 versus >100, the group with CAC levels exceeding 100 revealed significantly higher levels of OPG: 5.95 ± 2.53 vs 4.13 ± 2.62 pmol/L (P = 0.0133). These severely calcified patients were also significantly older (64 ± 12 vs 51 ± 16 years; P = 0.0003) (Table 2). None of the other parameters listed in Table 2 were significantly different between the two CAC groups. Patients with CAC Agatston score > 400 revealed OPG levels of 5.81 ± 2.67 (compared to 4.13 ± 2.62 pmol/L, p = 0.02). After stratifying patients according to presence or absence of AVC, the intergroup comparison revealed significantly lower sclerostin levels in patients without AVC (1.35 ± 0.73 vs 1.78 ± 0.84 ng/mL; P = 0.0311). There were no additional parameters with significant differences between the two AVC groups (Table 3).

**Table 2:** **Intergroup comparison for dialysis patients according to CAC level at baseline (*n*** **= 65)**

		**CAC <100**	**CAC >100 (65%)**
N	[n, (%)]	23 (35%)	42 (65%)
Gender (male)	[n, (%)]	10 (45%)	21 (50%)
Age	[yrs]	51 ± 16	64 ± 12
Time since first dialysis [mo]		87 ± 88	64 ± 68
BMI	[kg/m^2^]	26 ± 7	25 ± 5
CAC Agatston	28 ± 35	1014 ± 907	
AVC Agatson	14 ± 35	88 ± 266	
ucMGP	[nmol/L]	2199 ± 714	2423 ± 725
Fetuin-A	[g/L]	0.47 ± 0.12	0.45 ± 0.08
OPG	[pmol/L]	4.13 ± 2.62	5.95 ± 2.53
Sclerostin	[ng/mL]	1.52 ± 0.71	1.54 ± 0.86
CRP	[mg/L]	4.19 ± 6.35	8.05 ± 6.67
iPTH	[pg/mL]	119 ± 139	282 ± 396
BAP	[U/L]	29 ± 22	33 ± 28
AP	[U/L]	82 ± 46	89 ± 72
Total Ca2+	[mmol/L]	2.25 ±0.27	2.39 ± 0.29
Phosphorus	[mmol/L]	1.77 ± 0.53	1.83 ± 0.50
Albumin	[g/L]	39 ± 8	39 ± 4
Haemoglobin	[g/L]	109 ± 14	109 ± 27
S-Creatinine	[mg/dL]	9.7 ± 3.2	9.1 ± 2.6
S-Urea	[mmol/L]	82 ± 60	62 ± 61

**Table 3:** **Intergroup comparison for dialysis patients according to AVC level at baseline (*n*** **= 67)**

		**No AVC**	**Present AVC**
N	[n;%]	40; 60%	27; 40%
Gender (male)	[n;%]	19; 48%	13; 48%
Age	[yrs]	57 ± 16	63 ± 13
Time since first dialysis [mo]		76 ± 85	79 ± 81
BMI	[kg/m^2^]	25 ± 5	26 ± 7
CAC Agatston		507 ± 794	918 ± 938
AVC Agatson		0 ± 0	189 ± 340
ucMGP	[nmol/L]	2366 ± 791	2261 ± 589
Fetuin-A	[g/L]	0.46 ± 0.11	0.46 ± 0.07
OPG	[pmol/L]	4.96 ± 2.60	5.63 ± 2.819
Sclerostin	[ng/mL]	1.35 ± 0.73	1.78 ± 0.84
CRP	[mg/L]	7.77 ± 7.78	5.65 ± 4.55
iPTH	[pg/mL]	242 ± 350	190 ± 301
BAP	[U/L]	36 ± 30	27 ± 18
AP	[U/L]	91 ± 60	79 ± 66
Total Ca2+	[mmol/L]	2.39 ±0.21	2.27 ± 0.36
Phosphorus	[mmol/L]	1.76 ± 0.47	1.95 ± 0.63
Albumin	[g/L]	39 ± 7	39 ± 4
Hb	[g/L]	111 ± 14	108 ± 32
S-Creatinine	[mg/dL]	9.2 ± 2.9	9.8 ± 3.0
S-Urea	[mmol/L]	65 ± 59	81 ± 63

### Association between sclerostin and cardiovascular calcification

For both CAC and AVC, univariate and multivariate regression analyses were performed.

Depending on the P-values obtained in the univariate analysis (P ≤0.25 in Table 4), age, male sex, BMI, diabetes, OPG, iPTH, and ucMGP were included in the multivariable analysis for CAC. This multivariable analysis revealed that the presence of diabetes was significantly associated with CAC levels (Table 5, Agatston score). Similar results regarding the association with diabetes were obtained when analysing the volume CAC score and the mass CAC score (data not shown). The same model in n = 61 patients was applied after log transformation of CAC levels with age, male sex, BMI, calcium, OPG, CRP, and ucMGP entering the multivariate analysis. None of these univariate associations with log transformed CAC remained statistically significant after multivariate analysis.

**Table 4:** Univariate associate factor analysis regarding CAC Agatston score at baseline

		**95% limits of confidence**		
**Parameter**	**Estimate**	**Lower**	**Upper**	**p-value**
Age	8.7	−5.9	23	0.23
Gender male	411	−2.5	826	0.0358
Non-Smoking	−179	−800	442	0.3364
Non-Diabetics	−1145	−1661	−628	0.0012
Time ESRD	−0.6	−3.3	2.4	0.67
BMI	−25	−64	11	0.20
Fetuin-A	−38	−2306	14	0.97
Total Ca2+	55	−741	762	0.89
OPG	62	−13	141	0.13
CRP	4.6	−33	44	0.81
Sclerostin	77	−190	340	0.57
iPTH	0.3	−0.2	3.1	0.25
Pho4+	83	−329	458	0.70
ucMGP	0.4	0.1	0.7	0.0160
BAP	0.44	−7.0	8.0	0.91
Albumin	−7.5	−44	21	0.66

**Table 5:** Multivariate associate factor analysis regarding CAC Agatston score at baseline

**Parameter**	**Estimate**	**Lower**	**Upper**	**p-value**
OPG	46.62	−70	96	0.33
iPTH	0.42	−0.04	1.1	0.16
ucMGP	0.21	−0.1	0.5	0.16
Age	4.72	−236	2459	0.56
Gender male	267	−90	624	0.054
BMI	−22	−54	7.6	0.23
Non-diabetics	−1118	−1696	−540	0.0019

Regarding AVC (Agatston score) the univariate analysis (Table 6) resulted in inclusion of age, male sex, diabetes and serum sclerostin to the multivariable analysis. There was a significant association between serum sclerostin and the extent of AVC at baseline (P = 0.0170) (Table 7). Similar results regarding the association of serum sclerostin and AVC were obtained when anaylsing the volume AVC score and the mass AVC score (data not shown).

**Table 6:** Univariate associate factor analysis regarding AVC Agatston score at baseline

		**95% limits of confidence**		
**Parameter**	**Estimate**	**Lower**	**Upper**	**p-value**
Age	3.4	−0.4	7.4	0.08
Gender male	100	−15	215	0.098
Non-Smoking	−48	−220	123	0.54
Non-diabetics	−121	−281	39	0.0560
ime ESRD	−0.07	−1.0	0.6	0.84
BMI	1.29	−8.8	12	0.81
Fetuin-A	−29	−678	618	0.93
Total Ca2+	−75	−282	130	0.46
OPG	12	−9.5	36	0.27
CRP	−1.3	−12	9.5	0.81
Sclerostin	102	35	179	0.0058
iPTH	−0.04	−0.2	0.2	0.71
Pho4+	−50	−61	155	0.35
ucMGP	0.24	−0.1	0.1	0.56
BAP	−0.8	−3.5	1.4	0.51
Albumin	−2.7	−15	8.5	0.63

**Table 7:** Multivariate associate factor analysis regarding AVC Agatston score at baseline

**Parameter**	**Estimate**	**Lower**	**Upper**	**p-value**
Sclerostin	91	4.1	150	0.0170
Age	2.21	−1.0	7.2	0.28
Non-diabetics	−86	−250	79	0.08
Gender male	99	−20	217	0.15

### Sclerostin expression in aortic valves

**Figure 2:**
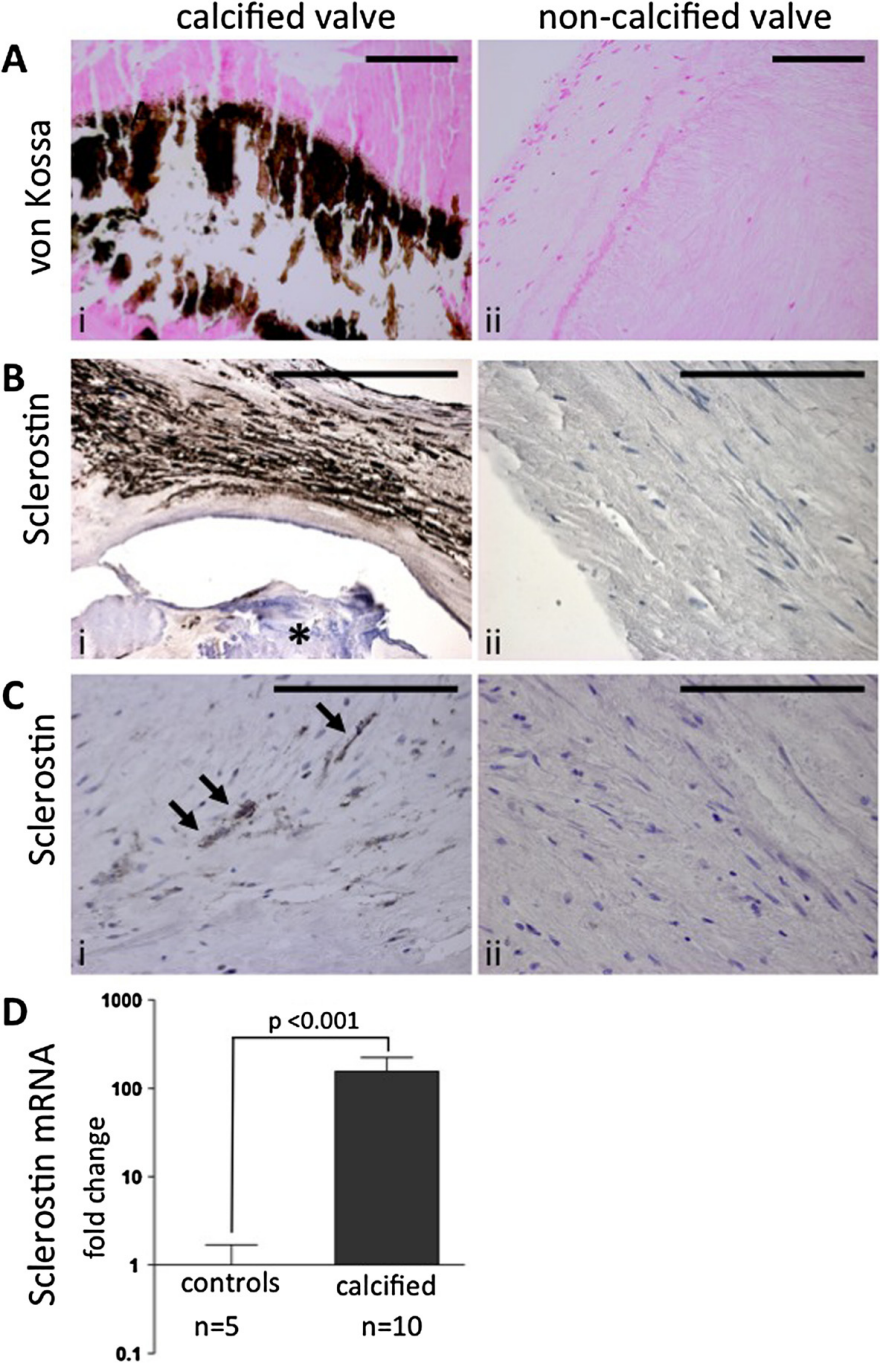
**Histological and immunohistochemical analyis of calcified and non-calcified valves from dialysis patients.** Von Kossa staining was strongly positive in calcified valves from dialysis patients **(Ai)** and completely negative in all non-calcified control valves **(Aii)**. Immunhistochemical staining for sclerostin revealed a strong expression in the calcified areas (arrows in **Bi**, calcification marked with asterisk) and a faint sclerostin expression in non-calcified areas (**Ci**, arrows) of the calcified valves from dialysis patients. The immunohistochemistry for sclerostin was negative in all non-calcified valves **(Bii, Cii)**. Qt-RT PCR for sclerostin expression showed a highly significant upregulation of sclerostin expression in the calcified valves compared to the non-calcified valves **(D)**. Gene expression in the non-calcified control valves was set as 1. All scale bars 50 ηm.

Immunohistochemical staining for sclerostin revealed sclerostin expression in all of the 10 calcified aortic valves while there was no sclerostin expression in the non-calcified control valves (Figure 2). The immunohistochemistry showed a strong sclerostin expression very close to the calcified regions (Figure 2B-i) but also a faint sclerostin expression in the non-calcified regions of the calcified valves from all of the haemodialysis patients (Figure 2C-i). In contrast, we did not observe any sclerostin expression in the non-calcified valves (Figure 2B-ii and C-ii). This finding was confirmed on the mRNA level via Qt-RT-PCR as we observed a highly significant upregulation of sclerostin mRNA in the calcified aortic valves of haemodialysis patients compared to non-calcified control valves (P <0.001) (Figure 2D).

## Discussion

The major findings of the present study were that in dialysis patients high serum sclerostin was associated with the extent of AVC and that in aortic valve tissue, sclerostin strongly co-localized with areas of calcification. In the last decade, numerous studies have investigated cardiovascular calcifications in adult haemodialysis patients (e.g. [6,21-23]). CVC clearly influences outcome in ESRD patient since previous studies could show an independent influence of baseline CVC [24] and its progression [2] upon mortality in these patients. Calcification of the aortic valves is an important part of the uraemic calcification syndrome. It has been previously shown that in patients without advanced renal failure there is a significant correlation between the amount of aortic valve calcification and the degree of aortic valve stenosis [25].

Previously published studies in dialysis patients regarding the association between various biochemical parameters and the degree of cardiovascular calcification do not show a homogeneous picture: Numerous circulating factors have been elaborated on so far as being associated with CAC or AVC but other studies failed to confirm these associations [1,6,22,26]. For example, the present study does not confirm an independent association between low levels of serum fetuin-A or high levels of OPG with CAC in contrast to a previous study by Moe et al. [12]. Our present data also differ from previous results that indicated a link between ucMGP levels and CAC in a smaller cohort of HD patients [27]. We can only speculate about the potential reasons. The different methodology of calcification detection and cohort size may play a role. Similarly, different patient characteristics (ethnic background, proportion of diabetics, dialysis vintage etc.) and moreover, preanalytical biomarker stability and assay characteristics of the investigated biomarkers clearly influence such associative investigations. In that respect it is noteworthy that especially fetuin-A serum measurements reveal meaningful differences depending on analytical procedures [28] and that previous data from our group indicating a link between fetuin-A in serum and the magnitude of VC had not been obtained by the TECOmedical assay used in the present study. So differences in serum fetuin-A assay set-ups most likely contribute to inconsistencies in terms of associations with the degree of calcification [12]. Similarly we need to point out that also previously reported serum sclerostin levels vary largely with different assays in hemodialysis patients: Data from our lab show a 45% lower circulating sclerostin concentration in hemodialysis patients measured with the TECOmedical assay compared to the Biomedica assay (V. Brandenburg, unpublished data).

Despite these limitations regarding biomarkers in uremic cardiovascular disease we see a convincing rationale to introduce sclerostin in CKD-mineral and bone disorder. Sclerostin strongly regulates bone metabolism. Genetically engineered animal models prove the role of sclerostin as a bone-suppressive factor [29,30]. Sclerostin knock-out mice were characterized by increased bone density and strength. Sclerostin exerts its osteo-suppressive actions via interference with the Wnt -signalling pathway [31]. The regulation of the Wnt-signalling pathway may contribute locally and/or systemically to bone and mineral disorders in CKD [15,32]. Recent study findings indicate that sclerostin is also involved in vascular disease. An *in vitro* and rodent study by Zhu et al. could show that sclerostin is upregulated in experimental models of vascular calcification [33]. We extend these findings for the first time to a potential linkage between sclerostin levels and AVC in humans with ESRD. The present data are in line with recent human study results indicating an association of sclerostin expression with non-uremic aortic valve calcification [34]. Therefore, sclerostin appears to be a promising future research target in CKD-MBD offering potential therapeutic perspectives [29,35].

Our data upon sclerostin expression in the vascular system stimulate the discussion about osteocyte involvement in vascular and valvular calcification processes. As previously speculated by Zhu et al. [33] the occurrence of sclerostin in the vasculature may indicate a terminal transdifferentiation from VSMCs towards a mature, osteocyte-like cell type. Zhu and coworkers investigated various osteocyte markers (e.g. DMP-1 and sclerostin) in different *in vitro* and *in vivo* calcification models. Indeed, VSMC sclerostin expression was stimulated by incubation in pro-calcific media and accordingly, sclerostin was detected in calcified aortas from ectonucleotide pyrophosphatase/phosphodiesterase 1 null mice [33]. Moreover, the different temporal course of osteocytic marker expression in osteoblasts compared to VSMC indicates that stimulated osteoblasts undergo this maturation process more readily than VSMC [33]. At that point, a crucial question arises whether local sclerostin production and systemic levels play a causal role in, or even trigger, the development CVC. Alternatively, sclerostin occurrence may just reflect a general phenotypic change of the vascular wall cells towards a bone-like phenotype. Most importantly, however, future longitudinal studies need to address the question whether sclerostin overexpression might even be vasculoprotective and anti-calcific via e.g. its indirect BMP-2 antagonistic activities.

It remains unclear why there was apparently no association between the amount of CAC and serum sclerostin levels. It is also unclear if the increase of serum sclerostin may be due to increased skeletal or extraosseous production, due to diminished renal clearance or both. The trial design does not allow drawing reliable conclusions of how CKD-MBD therapy (dialysis procedure, co-medication) influences sclerostin levels. At present, the biological meaning of the serum sclerostin differences between patients with AVC versus those without remains speculative. In conjunction with a recent publication by our group [34] serum sclerostin levels as measured with the present ELISA gradually increase with increasing cardiovascular disease burden: from controls without overt cardiovascular disease (0.76 ± 0.31 ng/mL), via non-renal patients with AVC (0.94 ± 0.45 ng/mL [34]), and via dialysis patients without AVC (1.35 ± 0.73 ng/mL) finally to dialysis patients with AVC (1.78 ± 0.84 ng/mL). Overall, we are aware that defining the exact association between serum sclerostin and CKD-MBD is challenging, and future human sclerostin studies in CKD-MBD should incorporate both vascular and osseous assessment [36,37]. The present data do not allow drawing firm conclusions whether sclerostin is within the aetiological pathway of uremic AVC development and whether baseline sclerostin levels are a risk factor for future CVC development.

We acknowledge several additional limitations of the present study including the cross-sectional nature, the small sample size of the MSCT patient group, the limited ability to adjust for all potential confounders as well as limited data availability in terms of treatment and comorbidities regarding the patients with aortic valve replacement. Moreover, we used only one assay type per parameter limiting the ability to universalize the associative data between biomarker with vascular calcification.

## Conclusion

In summary, we identified an association between both tissue sclerostin as well as serum sclerostin with aortic valve calcification in haemodialysis patients. Strength of our data is the homology between *ex vivo* (IHC) and *in vivo* (MS-CT) data. These findings add novel aspects to the well-described link between bone metabolism and cardiovascular health (bone-vascular axis) in uraemia. Further studies need to address the cellular origin of local sclerostin production and also investigate whether the appearance of sclerostin aggravates or rather slows down cardiovascular calcification processes.

## Abbreviations

ADPKD: Autosomal dominant polycystic kidney disease; (B)AP: (Bone) alkaline phosphatase; AVC: Aortic valve calcification; CAC: Coronary artery calcification; CKD: Chronic kidney disease; CKD-MBD: Chronic kidney disease – bone and mineral disorder; CRP: C-reactive protein; CVC: Cardiovascular calcification; ECG: Electrocardiogram; ELISA: Enzyme-linked immunosorbent assay; ESRD: End-stage renal disease; IHC: Immunohistochemistry; Hb: Haemoglobin; HD: Hemodialysis; (uc)MGP: (Uncarboxylated) matrix-gla protein; MS-CT: Multi-slice computed tomography; OPG: Osteoprotegerin; (i)PTH: (Intact) parathyroid hormone; Qt-RT-PCR: Quantitative reverse transcription polymerase chain reaction; RNA: Ribonucleic acid; SD: Standard deviation; VC: Vascular calcification.

## Competing interests

The authors declared that they have no competing interests.

## Authors’ contributions

VMB participated in developing the study design and conception of the study, recruitment of patients, performing the study and acquisition of data, laboratory analysis, immunohistochemistry, statistical analyses, and drafted the manuscript. VMB also revised the manuscript critically of intellectual content. RK participated in developing the study design and conception of the study, recruitment of patients, performing the study and acquisition of data, laboratory analysis, immunohistochemistry, statistical analyses, and drafted the manuscript. RK also revised the manuscript critically of intellectual content. RK participated in laboratory analysis, immunohistochemistry, statistical analyses, and drafted the manuscript. TK participated in laboratory analysis, immunohistochemistry, statistical analyses, and drafted the manuscript. LS participated in immunohistochemistry, statistical analyses, and drafted the manuscript. GM was responsible for performance of computed tomography calcification measurement, quality control and analyses. SH participated in recruitment of patients, performing the study and acquisition of data, laboratory analysis and drafted the manuscript. UG participated in recruitment of patients, performing the study and acquisition of data, laboratory analysis and drafted the manuscript. CD participated in statistical analyses, and drafted the manuscript. MK participated in developing the study design and conception of the study, recruitment of patients, performing the study and acquisition of data, laboratory analysis, immunohistochemistry, statistical analyses, and drafted the manuscript. MK also revised the manuscript critically of intellectual content. All authors read and approved the final manuscript.

## Pre-publication history

The pre-publication history for this paper can be accessed here:

http://www.biomedcentral.com/1471-2369/14/219/prepub
